# Supplementary material for: Solvent-Free Processed Cathode Slurry with Carbon Nanotube Conductors for Li-Ion Batteries
Source: Nanomaterials (Basel). 2023 Jan 12;13(2):324. doi: 10.3390/nano13020324 (PMC9863610; doi:10.3390/nano13020324)
Supplement: Supplementary file 1 [file nanomaterials-13-00324-s001.zip › nanomaterials-2137093-supplementary.pdf]

nanomaterials

Supporting Information for

Type of the Paper (Communication)

Solvent-Free Processed Cathode Slurry with Carbon Nanotube Conductors for Li-Ion Batteries

Gyori Park [1], Hyun-Suk Kim [2,*] and Kyung Jin Lee [1,*]

1 Department of Chemical Engineering and Applied Chemistry, Chungnam National University, Daejeon 34134, Republic of Korea
2 Department of Materials Science and Engineering, Chungnam National University, Daejeon 34134, Republic of Korea
* Correspondence: khs3297@cnu.ac.kr (H.-S.K.); kjlee@cnu.ac.kr (K.J.L.)

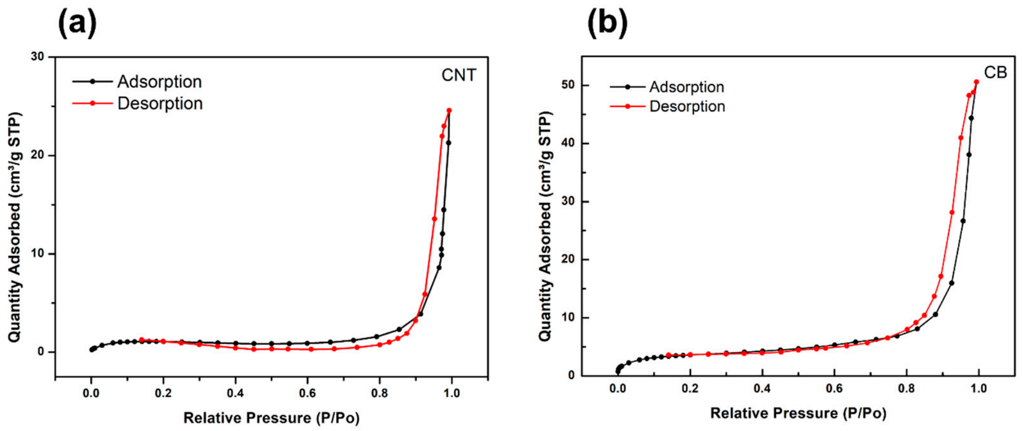

Figure S1. BET adsorption and desorption analyses graph of (a) CNT based electrode, (b) CB based electrode.

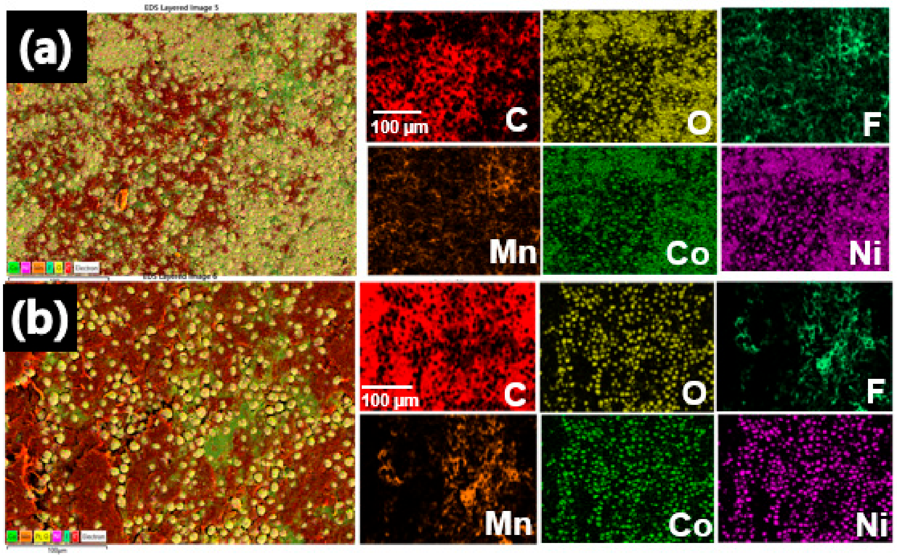

Figure S2. EDS map images of the dry process CNT based cathode (a) 90 : 5 : 5 (NCM811 : CNT : PTFE), (b) 70 : 25 : 5 (NCM811 : CNT : PTFE).

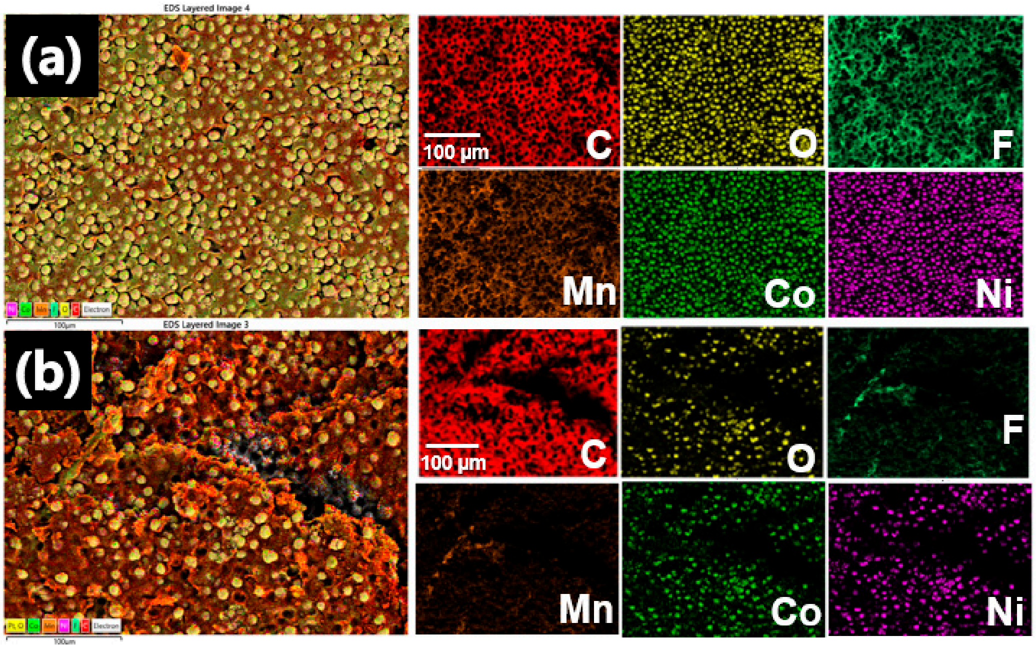

Figure S3. EDS map images of the dry process CB based cathode (a) 90 : 5 : 5 (NCM811 : CB: PTFE), (b) 70 : 25 : 5 (NCM811 : CB : PTFE).

Table S1. Detail information from Dry process CNT, Dry process CB based cathode sheet.

Ratio of NCM: Carbon/CB: binder	Electrode area (cm^2)	Thickness (µm) (Without Al foil)	Weight (g) (Without Al foil)	Electro density (g/cm^3)	Electrode Loading Level (mg/cm^2)
**Dry-CNT (NCM811 : CNT : PTFE)**					
90 : 5 : 5	1.54	200	0.1	3.25	65
85 : 10 : 5	1.54	210	0.11	3.4	71
**Dry-CB (NCM811 : CB : PTFE)**					
90 : 5 : 5	1.54	220	0.1	2.95	65
85 : 10 : 5	1.54	200	0.093	3.02	60
